# The effect of clinical guidelines on the utilisation of radiographs in chiropractic clinics in Denmark - an interrupted time series analysis

**DOI:** 10.1186/s12998-023-00518-9

**Published:** 2023-11-22

**Authors:** Pernille Schaldemose Reibke, Henriette Godskesen, Rikke Krüger Jensen, Simon D French, André Bussières, Henrik Wulff Christensen, Tue Secher Jensen

**Affiliations:** 1 Private chiropractic practice, Hillerød, Denmark; 2 Private chiropractic practice, Kalundborg, Denmark; 3 Department of Sport Science and Clinical Biomechanics, University of Southern Denmark, Odense, Denmark; 4 Chiropractic Knowledge Hub, Odense, Denmark; 5 Department of Chiropractic, Faculty of Medicine, Health and Human Sciences, Macquarie University, Sydney, Australia; 6 Département Chiropratique, Université du Québec à Trois-Rivières, Trois-Rivières, Québec, Canada; 7 Diagnostic Center – Imaging Section, Silkeborg Regional Hospital, Silkeborg, Denmark

**Keywords:** Radiography utilisation, Clinical guidelines, Chiropractor

## Abstract

**Background:**

In Denmark, chiropractors have a statutory right to use radiography and the government-funded national Health Insurance provides partial reimbursement. Danish National Clinical Guidelines recommends against routine use of imaging for uncomplicated spinal pain; however, it is not clear if clinical imaging guidelines recommendations have had an effect on the utilisation of spinal radiography. This study aimed to describe the utilisation rate of radiographs in Danish chiropractic clinics in the period from 2010 to 2020 and to assess the impact of clinical guidelines and policy changes on the utilisation of radiographs in Danish chiropractic clinics.

**Methods:**

Anonymised data from January 1st, 2010, to December 31st, 2020, were extracted from the Danish Regions register on health contacts in primary care. Data consisted of the total number of patients consulting one of 254 chiropractic clinics and the total number of patients having or being referred for radiography. Data were used to investigate the radiography utilisation per month from 2010 to 2020. An ‘interrupted time series’ analysis was conducted to determine if two interventions, the dissemination of 1) Danish clinical imaging guidelines recommendations and policy changes related to referral for advanced imaging for chiropractors in 2013 and 2) four Danish clinical guidelines recommendations in 2016, were associated with an immediate change in the level and/or slope of radiography utilisation.

**Results:**

In total, 336,128 unique patients consulted a chiropractor in 2010 of which 55,449 (15.4%) had radiography. In 2020, the number of patients consulting a chiropractor had increased to 366,732 of which 29,244 (8.0%) had radiography. The pre-intervention utilisation decreased by two radiographs per 10,000 patients per month. Little absolute change, but still statistically significant for Intervention 1, in the utilisation was found after the dissemination of the clinical guidelines and policy changes in 2013 or 2016.

**Conclusions:**

The proportion of Danish chiropractic patients undergoing radiography was halved in the period from 2010 to 2020. However, the dissemination of clinical imaging guidelines recommendations and policy changes related to referrals for advanced imaging showed little meaningful change in the monthly utilisation of radiographs in the same period.

## Background

Radiography has been used as a diagnostic tool amongst chiropractors since its incorporation in chiropractic clinical examinations in the early 1900s [1]. Radiography was routinely used for visualising the alignment of the spine with the purpose of identifying the biomechanical cause for spinal pain [1]. Since then, the understanding of the underlying concepts of spinal pain has moved towards a more patient-centred biopsychosocial model based on emerging scientific evidence [1]. Alongside this, guidelines advise against routine use of radiography in patients with spinal pain as there is no, or only limited, evidence suggesting a positive association with diagnosis, treatment choice or prognosis [2]. Despite this, a recent narrative review reported overuse of radiography in chiropractic practice, and wide geographical variation in radiography utilisation, ranging from 8–84% [3]. However, it is unclear if an overall downward trend in radiography utilisation is observed as some studies report a decrease while others report an increase in use [3–7].

To improve appropriate utilisation of radiography, clinical guidelines have been developed in recent years [3, 4]. In Denmark, the Danish health authorities continuously work on providing and updating national clinical guidelines based on scientific evidence to achieve high quality in healthcare at a national level [8, 9]. In 2013, the Danish Regions and the Danish Chiropractic Association (DCA) published clinical guidelines on diagnostic imaging of the musculoskeletal system in collaboration with radiology specialists [10]. Before 2013, no updated Danish guidelines for diagnostic imaging of the musculoskeletal system was available. The key purpose of the guidelines was to reduce inappropriate imaging procedures, including double examinations [10]. The guidelines were primarily aimed at chiropractors; however, they are also recommended for use by other clinician-types who utilise diagnostic imaging [10]. In 2015 and 2016, the Danish Health Authority published four National Clinical Guidelines for the management of patients with neck and low back pain with and without radiculopathy [8, 11]. While these guidelines advised against the routine use of diagnostic imaging to patients with recent onset of non-specific low back pain with or without radiculopathy, no mention is made regarding imaging use for patients with recent onset neck pain.

Clinical guidelines are designed to improve quality and reduce variation in the management of patients by changing the clinicians’ behaviour [12]. However, translating guidelines into clinical practice remains a challenge due to factors such as; clinicians relying on past experience and clinical judgment over the use of guidelines, maintaining a positive patient-clinician relationship through imaging referrals, and limitations from the clinicians’ side to implement guidelines recommendations, such as lack of time and knowledge of the guidelines [13]. In a systematic review, Jenkins et al. investigated whether utilisation of diagnostic imaging followed guidelines globally and concluded that inappropriate imaging is common in low back pain management, including both overuse in patients where imaging is not indicated as well as underuse of imaging when it is indicated [3]. International studies have investigated the effect of clinical guidelines on the use of radiographs in chiropractic practice. Bussières et al. reported an immediate reduction in imaging claims after introducing web-based imaging guidelines for uncomplicated low back and neck pain in the United States [7]. Through a survey amongst Australian chiropractors, Jenkins et al. concluded that “A poorer awareness of guidelines is associated with an increase in the reported likelihood of use, and the perceived usefulness of radiographs for low back pain, in clinical situations that fall outside of current guidelines” [14]. Fine et al. investigated the effect of restricting diagnostic imaging reimbursement in Ontario and reported a decrease in x-rays ordered by family physicians in the years following the policy change [4]. A more recent randomised controlled trial, including physiotherapists and chiropractors who were assigned either to a tailored, multi-faceted intervention based on the Australian clinical practice guideline for acute back pain or to passive dissemination of the guideline, reported that the intervention group clinicians were more likely to intend to adhere to the guideline for X-ray referral, although there was no difference between the two intervention groups with respect to the proportion of patients referred for radiographs [15].

To our knowledge, no existing articles have reported on the effect of the guidelines on the utilisation of radiographs in a Danish chiropractic setting. The objectives of this study were therefore to: (1) describe the utilisation rate of radiographs in Danish chiropractic clinics from 2010 to 2020; and, (2) assess the impact of disseminating Danish clinical guidelines in 2013 and 2016 on the utilisation of radiographs in Danish chiropractic clinics.

## Methods

### Design

This study is a retrospective quasi-experimental design using interrupted time series analysis to assess the potential impact of implementation of clinical guidelines in 2013 and 2016.

### Setting

The Danish healthcare system is primarily publicly funded [16]. Taxes finance approximately 83% of all Danish healthcare expenses, including free access to hospitals, general practitioners, as well as partial reimbursement of prescribed medications, physiotherapy, and chiropractic services [17]. Approximately every third year the DCA negotiates an agreement with the Danish health authorities (The Danish Regions’ Board for Wages and Tariffs) on behalf of the chiropractic profession [18]. The agreement includes the conditions under which chiropractors practice, as well as terms and quantity of reimbursement for chiropractic, which in 2022 was approximately 22% for radiographs. All eligible reimbursements to chiropractic clinics, including imaging, are registered by Danish Regions based on billing codes [19].

### Data collection

Anonymised data from January 1st, 2010, to December 31st, 2020, were extracted from a register at the Danish Regions. Data comprised the monthly and yearly number of all unique patients who consulted a chiropractor and the number of unique patients who had radiography. Data on all billing codes for radiography per month and year were identified and extracted.

### Study population

To receive reimbursement, and thereby be registered in the billing code registry, a chiropractic clinic must be a part of the Danish collective agreement for chiropractors. In September 2014 there were 264 clinics in Denmark whereof 228 clinics (86.4%) were a part of the Danish collective agreement for chiropractors [20]. In 2021 this number had increased to 254 of 278 clinics (91.3%) [21]. The Danish collective agreement for chiropractors requests that all chiropractic members should have radiographic equipment available, either at their own clinic or through an agreement with another chiropractic clinic or a hospital department [19]. The study population therefore comprised patients who attended one of the chiropractic clinics in Denmark that were part of the collective agreement from 2010 to 2020 [21].

### Variables

Data were categorised by age groups and sex. As a unique patient can occur once every month over a calendar year, the monthly rates of unique chiropractic patients would be lower than compared to yearly rates. The number of radiographs were extracted and summarized from the following billing codes: Primary radiographic examination of the clinician’s own patient (billing code 2014); Primary radiographic examination by referral from other chiropractor (billing code 2015); and, Supplementary radiographic examination (billing code 2020) [19].

### Interventions

In this study we chose to analyse the impact of two periods of dissemination and implementation of the clinical guidelines and collective agreements in the study period (2010–2020) that may have had an impact on chiropractors utilisation of radiographs: (1) The Danish clinical guidelines for diagnostic imaging of the musculoskeletal system from 2013 [10], and the collective agreement between DCA and Danish Regions from January 2014 [22]; and (2) the four Danish National Clinical Guidelines for managing neck and low back pain published in 2015 and 2016 [8, 11].

The clinical guidelines for diagnostic imaging of the musculoskeletal system were published and made available online in May 2013 (revised in 2014 online only) [10]. All members of the DCA and the Danish Society of Radiology received a printed version in May 2013. On January 1st 2014, a new three-year collective agreement between the Danish Regions and the DCA became effective. The new agreement included an article (§ 8) that allows Danish chiropractors to refer patients with musculoskeletal problems for CT- or MRI-scans [23] based on the recommendations from the Danish imaging guidelines. The imaging guidelines and the new agreement were presented and discussed at workshops for chiropractors in the five Danish regions in January 2014, organised by The Chiropractic Knowledge Hub (former NIKKB), thus here considered as one intervention.

The four national clinical guidelines for managing neck and low back pain were published in the period from May 2015 to November 2016: (1) National clinical guidelines for non-surgical treatment of cervical radiculopathy (May 2015) [11, 24] specifies in the background section that MRI should be performed in cases of suspicion of serious pathology or if surgery is considered; (2) National clinical guidelines for non-surgical treatment of lumbar radiculopathy (January 2016) [8, 25] recommends against routine use of MRI; (3) National clinical guidelines for non-surgical treatment of recent onset of low back pain (June 2016) [8, 9] recommends against routine use of MRI or radiography; and, (4) National clinical guidelines for non-surgical treatment of recent onset neck pain (November 2016) [11, 26] does not mention imaging. The four guidelines were (and still are) available on the website of the Danish Health Authority. Members of the DCA were made aware of the guidelines by newsletters from DCA and in the DCA professional journal “Kiropraktoren”. The guidelines were also presented at the annual meetings of the DCA in 2015 and 2016, in online DCA newsletters and published in a peer reviewed journal [8, 11]. The Chiropractic Knowledge Hub organised workshops in each of the five Danish regions and at the annual meeting of the DCA in December 2016 to further disseminate knowledge about the guidelines. Although not all of the clinical guidelines include specific recommendations regarding radiography, we chose to include them on the basis that the utilisation of diagnostic imaging, including radiography, was discussed at the DCA meetings and the workshops.

Considering that passive diffusion, such as printed educational material and online publications of guidelines, is associated with a small change in clinical behaviour only [7], a more active and clinician engaging approach, e.g. workshops, is considered a more effective implementation strategy [27]. Therefore, we chose to use the end dates for the workshops as the intervention date in the statistical analysis (see below): *January 2014* for the implementation of the imaging guidelines and the 2014 collective agreement and *December 2016* for the clinical guidelines on neck and low back pain.

### Analysis

To describe the utilisation rate of radiographs in Danish chiropractic clinics, yearly data on unique patients having radiography was used as the numerator and the number of unique annual chiropractic patients as the denominator to calculate a percentage. The use of proportions eliminates the impact of a change in numbers of chiropractic clinics, chiropractic patients and seasonal trends.

To assess the impact of the two interventions in January 2014 and December 2016, respectively, on the utilisation of radiographs in Danish chiropractic clinics, an interrupted time-series (ITS) analysis was performed using monthly data. The monthly proportion of patients having radiography was calculated using the number of unique patients having radiography as the numerator and the total number of unique chiropractic patients as the denominator. For interpretation purposes, we multiplied the proportion by 10,000. The significance of a change in slope and immediate level change of the slope before and after the two interventions were calculated using segmented regression analysis. The model requires a minimum of eight time points before and after an intervention [28] and therefore only monthly data were included in the analysis. The level of statistical significance was set at 5%. All statistical analysis was performed in STATA 16 (Stata Corp LLC, Collage Station, USA). As time-series data are typically autocorrelated, we took a conservative approach and used linear regression analysis with autoregressive errors. We used the ‘itsa’ command in STATA, which uses the ordinary least squares (OLS) model with Newey-West standard errors to deal with autocorrelation and heteroskedasticity.

Given that the interventions were implemented over a period of time rather than at a single time point, we conducted a sensitivity analysis. To account for the time lag between guideline publication and implementation in practice, we defined a ‘phase-in’ period for each of the two interventions and the data points for these periods were excluded from the sensitivity analysis. For Intervention 1, we censored the data between May 2013 and January 2014, and for Intervention 2, we censored the data between May 2015 and November 2016. In addition, the time period used in this analysis overlaps with the COVID-19 pandemic, which could act as a concurrent event and potentially affect the outcome. Therefore, we performed a sensitivity analysis censoring the period from March 2020 onwards to account for potential confounding effects. Finally, the post-analysis autocorrelation function (ACF) and partial autocorrelation function (PACF) plots indicated risk of seasonality and we therefore performed a sensitivity analysis controlled for seasonality using seasonal differencing adjustments and testing for stationarity using Dickey-Fuller test.

## Results

### Population

In total, 336,128 unique patients consulted a chiropractor in 2010 of which 55,449 (15.4%) had radiography. In 2020, the number of patients consulting a chiropractor had increased to 366,732 and of these 29,244 (8.0%) had radiography.

**Fig. 1:**
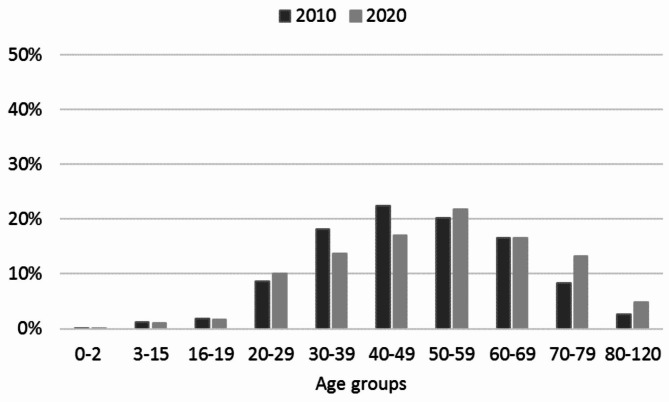
Distribution by age groups of patients having radiography in 2010 and 2020 in Danish chiropractic clinics

Data from 2010 to 2020 of patients having radiography were divided into age groups of which the largest in 2010 was 40–49 years and 50–59 years in 2020. The percentage of each age group in 2010 and 2020 is shown in Fig. 1.

More than half of those who consulted a chiropractor in 2010 were women (55.7% in 2010 and 54.5% in 2020). Of patients undergoing radiography, the proportion of women was 51.1% in 2010 and 48.6% in 2020.

#### Utilisation rate of radiographs

**Fig. 2:**
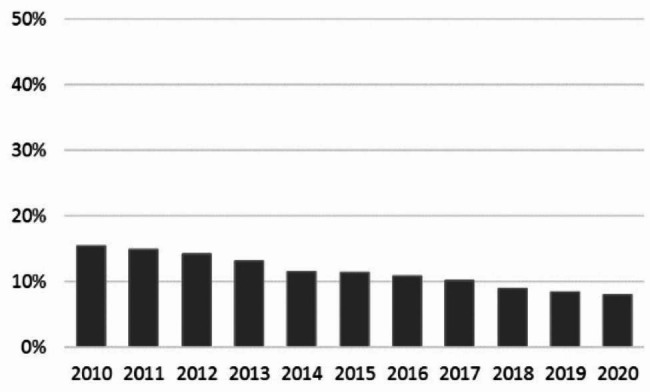
Utilisation rate of radiographs in Danish chiropractic practice from 2010 to 2020

There was an overall decrease in the utilisation of radiographs in Danish chiropractic practice from 15.4% to 2010 to 8.0% in 2020, corresponding to a yearly decrease of 0.8% (95% CI 0.68–0.85). (Fig. 2)

### Impact of clinical guidelines

When analysing the utilisation rate on a monthly basis, there was a decrease in the overall utilisation of radiographs in Danish chiropractic practice from 5.2% of patients having radiographs in 2010 to 2.7% in 2020, with a statistically significant average monthly decrease of 0.02% (95% CI 0.019–0.021), corresponding to a decrease of two radiographs per 10,000 patients per month.

#### First intervention

**Fig. 3:**
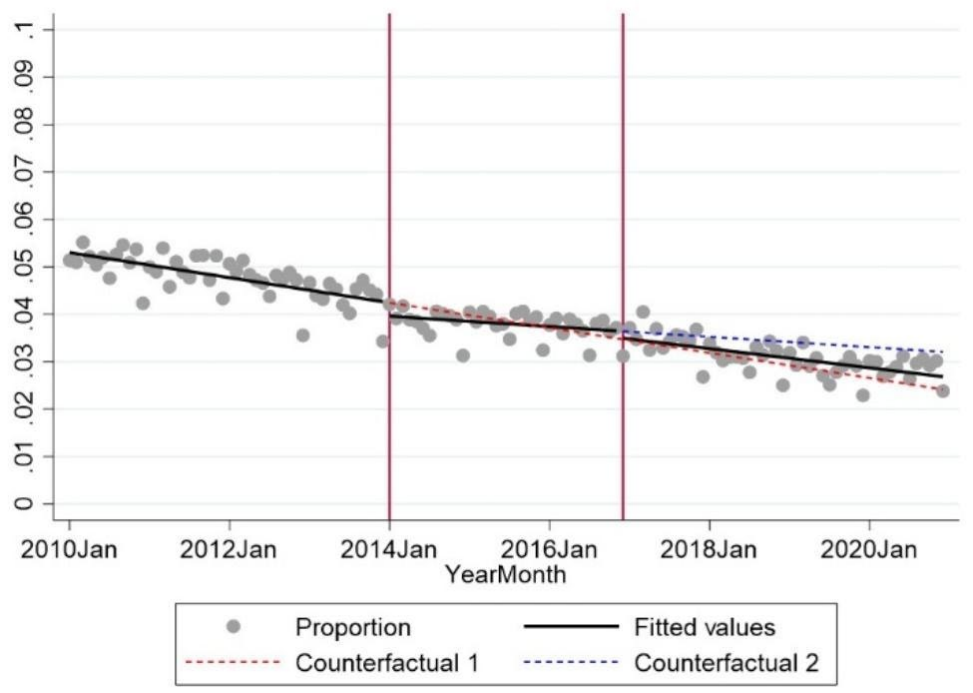
Monthly data from 2010 to 2020 on the proportion of chiropractic patients having diagnostic imaging Note: Vertical red lines mark the interventions in January 2014 and December 2016. The x-axis is in months with ‘2010Jan’ representing January 2010 etc. The y-axis presents the proportion (0–1) of patients who received radiographs per month and is reduced to 0-0.1 for clarity

From 2010 to 2013 (pre-intervention) there was a monthly decrease of 2.2 radiographs per 10,000 patients (p < 0.001). After the first intervention there was a statistically significant level change of 28.4 fewer radiographs per 10,000 patients (p = 0.04) and a statistically significantly increase in the monthly utilisation (slope) of 1.3 radiographs per 10,000 patients (p = 0.006). (Fig. 3; Table 1)

**Table 1:** Analysis of the utilisation of radiographs (number of radiographs per 10,000 patients) in chiropractic practice in the period from 2010 to 2020

	Coefficient	95% CI		P-value
Pre-intervention slope	-2.20	-2.87; -1.53		**< 0.01**
**Intervention 1 (January 2014)**				
Level change after first intervention	-28.37	-55.18; -15.48		**0.04**
Slope change after first intervention	1.30	3.86; 0.00		**< 0.01**
Slope after first intervention	-0.91	-1.60; -0.21		**0.01**
**Intervention 2 (December 2016)**				
Level change after second intervention	-14.71	-35.02; 5.59		0.15
Slope change after second intervention	-0.78	-1.64; 0.01		0.08
Slope after second intervention	-1.69	-2.21; -1.17		**< 0.01**

P-values < 0.05 are marked in bold

#### Second intervention

Following the second intervention, there was a level change of 14.7 fewer radiographs per 10,000 patients and a further decrease in the utilisation (slope)of 0.78 radiographs per 10,000 patients, although both estimates were not statistically significant (p = 0.15 and p = 0.08, respectively) as compared to the time period between the two interventions. (Fig. 3; Table 1)

### Sensitivity analysis

Sensitivity analyses were performed to account for (1) seasonality, (2) pandemic period, and (3) interventions as time periods instead of single time points.

Seasonality: As the post analysis autocorrelation function (ACF) and partial autocorrelation function (PACF) plots indicated seasonality, we adjusted the dataset for this. The analysis of the adjusted dataset resulted in only minor changes of all the estimates. However, these changes were not significant (overlapping 95% CIs) and the new analysis did not change the overall results as compared to the original analysis, data not shown.

COVID-19 pandemic period: Censoring data points for the period of the pandemic (March 2020 to December 2020) did not change the estimates significantly (overlapping 95% CIs) as compared to the original analysis, data not shown.

Interventions as periods instead of time points: As the interventions were implemented over a period rather than at a single time point, we also analysed the data consoring between the start of the interventions to when they were fully implemented, e.g. between May 2013 and January 2014 for Intervention 1 and between May 2015 to November 2016 for Intervention 2). The new analysis did not change the estimates significantly (overlapping 95% CIs) as compared to the original analysis, data not shown.

## Discussion

### Main findings

The yearly utilisation rate of radiographs in Danish chiropractic clinics decreased significantly from 15.4% to 2010 to 8% in 2020. However, the dissemination of Danish clinical guidelines and the collective agreement that gave Danish chiropractors the option to refer patients for advanced diagnostic imaging did not change the utilisation of radiography considerably. The first intervention showed a statistically significant level change and slope change; these changes corresponded to 28 fewer radiographs per 10,000 patients per month and an increase of 1.3 radiographs per 10,000.

Using the results from the analysis (Fig. 3) we estimated the monthly utilisation by December 2020 for the pre-intervention period and after each of the two interventions. If none of the interventions had been introduced (Counterfactual 1), the average monthly utilisation would have been 2.4% in December 2020. With both of the interventions in effect (Fitted line), the average monthly utilisation of radiographs was in reality 2.7%. If the second intervention had not been introduced (Counterfactual 2), the average monthly utilisation in December 2020 would have been 3.2%. This corresponds to 3 extra radiographic examinations per 1,000 patients for the scenario where the second intervention would not have been introduced compared to the current scenario. With the introduction of the second intervention, the difference between the pre-intervention utilisation and the actual (Fitted) monthly utilisation for December 2020 was 5 radiographic examinations less per 1,000 patients.

### The utilisation rate of radiography

The reduction of about 50% in the rate of radiography usage among Danish chiropractors from 2010 to 2020 should be viewed by considering that a substantial decrease was also present in Denmark in the years prior to 2010. In a study from 2002 to 1,595 chiropractic patients, 27% had radiography taken on the day of their visit with more radiographs taken with increasing age and longer duration of symptoms [6].

With the current decrease in utilisation presented in this study, it is possible that radiographs will no longer be used in Danish chiropractic practice within a decade or two. Although the ’optimal’ imaging rate of patients seen in chiropractic practice is unknown, the decreasing usage of diagnostic imaging would potentially level out at some point as there are absolute indications of imaging, including radiography. For low back pain, the prevalence of serious pathology has been suggested to range between 0.9 and 4.5% [29, 30]. If the rate of radiography becomes too low, there is a risk that relevant pathologies could be missed. However, the ‘correct’ use of imaging must be related to guidelines-informed indications, where patient symptoms and clinical findings are taken into account, rather than an arbitrary utilisation rate [3].

The decreasing use of radiographs in Denmark could potentially be caused by factors other than the publication and implementation of clinical guidelines. The change in the undergraduate training of Danish chiropractors, as well as increased knowledge about and access to other diagnostic imaging modalities, such as MRI, could have influenced the utilisation of radiography. The Danish chiropractic education was established in 1994 as a university based five-year education at the Faculty of Health Sciences, University of Southern Denmark (SDU), and the majority of Danish chiropractors (60% in 2020) are now graduates from SDU [31]. SDU education is evidence-based and clinical subjects are taught in accordance with current clinical guidelines. It may be that the decrease in the utilisation seen in this study could have been influenced by a generation of Danish chiropractors for whom radiography is viewed as one among several imaging modalities and not a “one size fits all”.

When analysing the study sample, we found an increase in age for chiropractic patients having radiography from 2010 to 2020. First, it is notable that the study period is 11 years. Therefore, the same group of patients most commonly receiving radiography is the same in 2010 (40–49 years of age) and 10 years later, in 2020, (50–59 years of age). This is in accordance with Kiel et al. [32] who found that having had previous imaging was one of the predictors for patients with low back pain expecting imaging.

#### Impact of interventions

The implementation of Danish guidelines and policy changes investigated in the present study was shown to be less effective when compared to similar interventions. Bussières et al. reported a statistically significant drop after publication of web-based guidelines known among clinicians known to have high radiography imaging rates [7]. However, the Danish guidelines were also published online and was combined with additional active implementation tools, i.e. presentations and workshops, but did not provide as large a drop in utilisation rates. Fine et al. found that restrictions on reimbursement for imaging on uncomplicated low back pain was also effective [4]. During the study period (2010–2020), the reimbursement for radiographs in Danish chiropractic clinics was constant at 22.2%. Reimbursement restrictions may be an option in Denmark, if there is evidence for a need of further decrease in the utilisation.

The primary aims of the Danish national clinical guidelines on neck and low back pain were to promote more evidence-based management of patients with spinal pain, including imaging. The present study found that, although there was a large and significant decrease in the proportion of patients with radiographs over the study period, the interventions themselves did not seem to have an important effect on the change of the use of radiography in chiropractic practice. The effect of interventions in relation to behaviour change depend on its dissemination and implementation. Therefore, it can be helpful to evaluate facilitators and barriers. The publishing of the guidelines was facilitated by sending out printed versions to members of the DCA and the guidelines were presented at two annual meetings and made easily accessible online. Moreover, the Chiropractic Knowledge Hub (former NIKKB) engaged the members of DCA in a more active manner by setting up regional workshops which had a considerable reach. Potential barriers could include misalignment with patient expectations or the chiropractors’ habits, experience or diagnostic confidence [33]. Also, the guidelines recommendations may be perceived by the chiropractor as too extensive to remember in daily practice. Since the guidelines did not statistically significantly affect the utilisation of radiography it seems as if facilitators did not overcome the barriers.

Based on the considerations above, one may argue, that the decrease in the utilisation is a continuation of the focus from Danish chiropractors on both technical and clinical aspects of imaging and that the guidelines messages were consistent with other initiatives and therefore could have made a collective contribution to the decreased imaging rates overall. This is a process that started before the turn of the century with the focus of integrating chiropractors in the public health care system. With regards to imaging, national and international publications, such as a Danish quality assurance report on low back pain and chiropractic [34], which had an extensive chapter on imaging, and the “Diagnostic imaging practice guidelines for musculoskeletal complaints in adults” by Bussières et al. (2007 and 2008) are likely to have had a positive impact on the decrease of radiography in Danish chiropractic care.

### Methodical limitations and strengths

One major limitation of the study is that the interventions were not well-defined in time and were not defined before data collection (pre-hoc). Also, the data available were limited to the utilisation of radiography with no knowledge of the patients’ signs and symptoms or the chiropractors’ attitudes and beliefs towards diagnostic imaging. Hence, factors beyond what is described in the interventions may have influenced chiropractic use of diagnostic imaging.

This study includes data from patients of all ages, including infants and children, although the clinical guidelines only apply to adult patients. However, as the increase of patients aged 0 to 17 years, increased by less than 1%, from 8.7% to 2010 to 9.4% in 2020 (data not shown), we do not consider this to have had an effect on the interpretation of the results.

The included data does not distinguish between radiographs of different regions of the spine or extremities. The clinical guidelines from 2015 to 2016 only concern radiography of the cervical and lumbar spine regions. Therefore, conclusions of the effects of the interventions for different anatomical regions cannot be drawn from this study.

This study does not include data from all Danish chiropractic clinics as a smaller proportion of clinics are not part of the Mutual Agreement with Danish Regions. Nevertheless, since the vast majority (> 90%) of the Danish chiropractic clinics are included, the study’s representativeness is considered acceptable [20, 21, 31].

Furthermore, not all chiropractic clinics have their own radiographic equipment and therefore refer to other clinics or hospitals [19]. The radiographic examinations performed at hospitals are not included and the proportion of radiography referrals could therefore be underestimated.

This study also has several strengths. ITS analysis is one of the strongest quasi-experimental methods which considers secular trends and the ability to evaluate both wanted and unwanted effects of interventions [28]. To perform the analysis, a minimum of eight time points is required before and after each intervention. This study has 131 time points with at least 36 time points before and after each intervention. The relatively large amount of data and the long study period of 10 years reduces the uncertainty and minimises the chance of secular trends affecting the result. Furthermore, the study sample of this study is large (n > 88,000 patients per time point/month), which is only possible due to the Danish national registries.

### Perspectives

In 2020, radiographic usage amongst Danish chiropractors was 8%, which can be considered fairly low when the prevalence of serious pathology is taken into account. Future research is needed to investigate if patients seen in chiropractic practice have clinical signs and symptoms that are in accordance with indications for imaging in cross-sectional studies and if these signs and symptoms really are predictors for serious pathology in longitudinal studies. Also, future research should investigate and identify patient and clinician factors that may be related to inappropriate utilisation, both over- and underuse, with regards to clinical guidelines.

## Conclusion

This study found a continued significant decrease in Danish chiropractors’ utilisation of radiography which were reduced by half from 2010 to 2020. However, the implementation of clinical guidelines and policy changes relevant for diagnostic imaging within the same period showed little meaningful change on the utilisation. Future research should investigate and identify patient and clinician factors that are related to inappropriate utilisation of imaging, both over- and underuse, with regards to clinical guidelines.

## Data Availability

The data that support the findings of this study are available from Danish Regions [16] but restrictions apply to the availability of these data, which were used under license for the current study, and so are not publicly available. Data are however available from the authors upon reasonable request and with permission of Danish Regions.

## List of abbreviations

CI: Confidence interval
CT: Computed Tomography
DCA: Danish Chiropractic Association
ITS: Interrupted time series
MRI: Magnetic Resonance Imaging
NIKKB: Nordic Institute of Chiropractic and Clinical Biomechanics
SDU: University of Southern Denmark
